# Identification of pathogenic *C9orf72* hexanucleotide repeat expansion in a Chinese patient with frontotemporal dementia: A case report

**DOI:** 10.1111/cns.13639

**Published:** 2021-03-31

**Authors:** Yan‐Yan Xue, Zhi‐Ying Wu, Hong‐Fu Li

**Affiliations:** ^1^ Department of Neurology and Research Center of Neurology in Second Affiliated Hospital, and Key Laboratory of Medical Neurobiology of Zhejiang Province Zhejiang University School of Medicine Hangzhou China

Dear Editors,

Frontotemporal dementia (FTD) is a common form of dementia, characterized clinically by behavioral disturbance, progressive cognitive impairment, and language impairment owing to the degeneration of the frontal and temporal lobes. The discovery that abnormal GGGGCC hexanucleotide repeat expansions (HRE) in *C9orf72* was a common cause of FTD and amyotrophic lateral sclerosis (ALS) fueled and speeded up the research in *C9orf72*‐related ALS and FTD worldwide.^1^, ^2^ This pathogenic expansion could cause disease by loss of normal function of C9orf72 protein, RNA toxicity, and dipeptide‐repeat proteins aggregates.^3^, ^4^ Although the mechanisms for different phenotypes of *C9orf72* HRE have not been well unveiled, the proteomic cerebrospinal fluid analyses and imaging studies provided potential biomarkers for *C9orf72*‐linked FTD and ALS.^5^, ^6^ Previous studies showed the most common symptoms of *C9orf72*‐linked FTD were behavioral disinhibition and psychiatric disorders.^7^, ^8^, ^9^ Notably, 60% of *C9orf72*‐related FTD patients developed motor neuron disease symptoms during follow‐up.^9^ *C9orf72* HRE was the leading genetic cause of ALS and FTD in Caucasian populations, accounting for 21.7%‐47.0% in familial ALS, 13.8%‐48.1% in familial FTD, 4.1%‐21.1% in sporadic ALS, and 2.2%‐18.8% in sporadic FTD.^10^, ^11^ However, the *C9orf72* HRE was rare in the Asian population, which could only explain around 0.4% ALS in Japan, 3.5% family ALS, and 0.5% sporadic ALS in Chinese populations.^12^, ^13^ The prevalence of *C9orf72*‐related FTD was not clear in China, which calls for a large‐scale multicenter study. To date, only two Chinese cases of *C9orf72*‐related FTD patients were reported.^14^, ^15^ We herein reported a 41‐year‐old female FTD Chinese patient, who had a familial history of ALS and carried pathogenic *C9orf72* HRE (>93, normal <30).

In March 2017, a 41‐year‐old female was admitted to the Department of Neurology with the complaint of progressive speech disturbance and memory impairment for one year. During this time, she presented hesitant speech and word‐finding difficulty, accompanied by poor articulation. Also, she had difficulties in recalling. The neuropsychological assessment demonstrated cognitive dysfunction, shown as 14/30 points in Mini‐Mental State Examination, 1/3 point in Clinical Dementia Rating Scale, and 38/80 points in Activity of Daily Living Scale. The neuropsychiatric inventory test revealed intact psychological status. Neurological examinations were unremarkable. Notably, her father and two uncles were diagnosed with ALS in their 50s and passed away several years later (Figure 1A). Laboratory tests and electroencephalogram (EEG) showed no obvious abnormality. Cerebrospinal fluid examinations including basic constituents, Aβ_1–42_, total tau, and phosphorylated tau were within the normal range. Brain Magnetic resonance imaging (MRI) revealed significant frontal and temporal lobe atrophy (Figure 1B). Electromyography (EMG) test revealed distal peripheral neuropathy.

**FIGURE 1:**
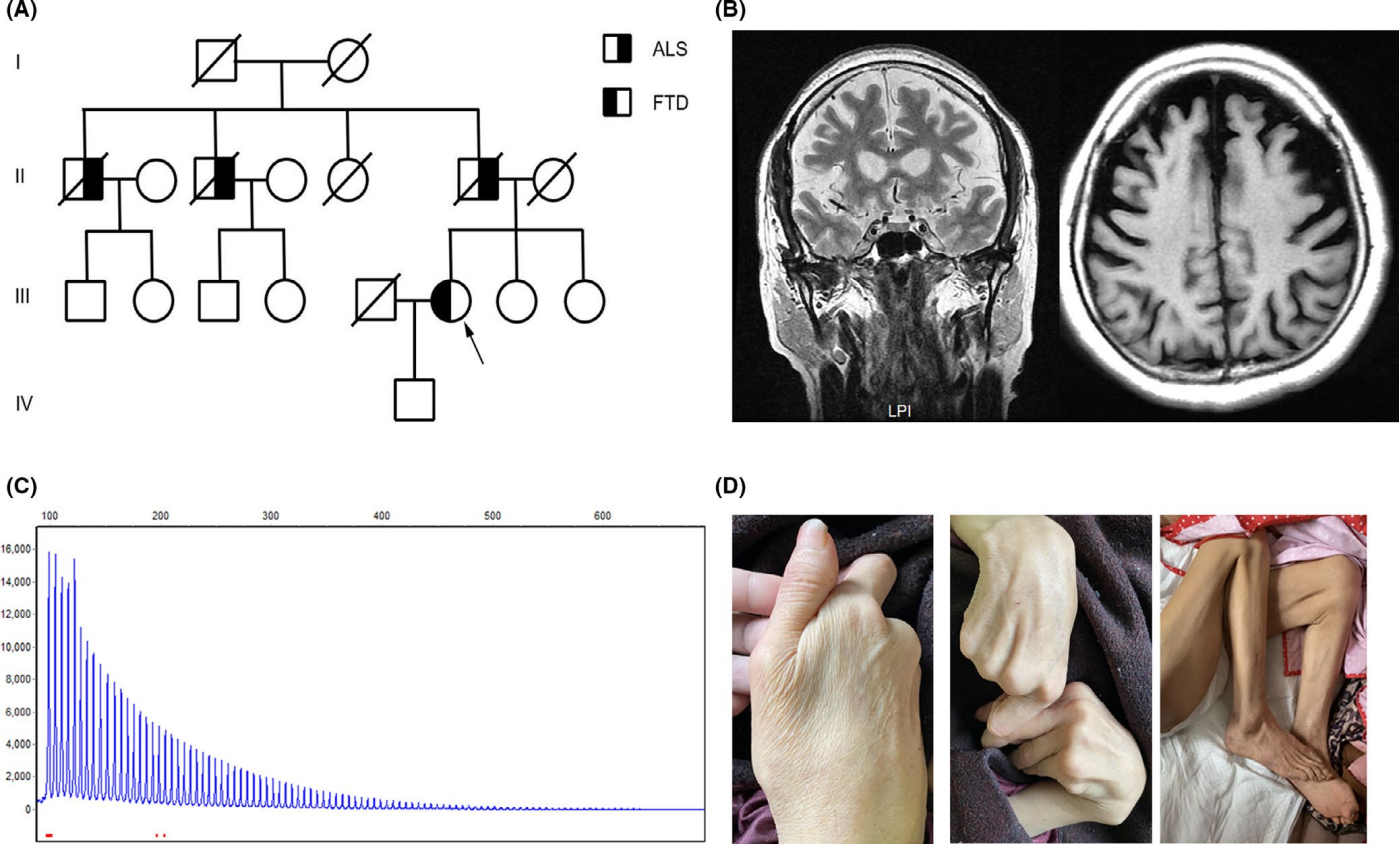
The genetic and clinical information of the patient carrying the hexanucleotide repeat expansion in *C9orf72*. (A) The pedigree of the family; (B) Brain MRI showed frontal and temporal lobes atrophy; (C) The electropherograms of the PCR products of repeat‐primed PCR reactions investigating the hexanucleotide repeat expansion in *C9orf72*; (D) Neurological examinations revealed severe muscle atrophy at a follow‐up visit

In consideration of her obvious family history, we performed the whole exon sequencing and repeat‐primed PCR to screen GGGGCC repeat expansion in the *C9orf72*. The genetic investigation revealed more than 93 times repeat expansions (normal <30) in *C9orf72* (Figure 1C).

Two years later, the patient’s cognition was further declined. Moreover, the patient exhibited limbs weakness, progressive muscular dystrophy, and dysphagia. Neurological examinations revealed hypermyotonia, fasciculations, hyperreflexia in the affected limb, muscle weakness, and severe muscle atrophy (Figure 1D). These symptoms could be explained by *C9orf72*‐related ALS, but the patient denied EMG examination.

We also screened the *C9orf72* HRE in other 36 FTD patients and reviewed previous studies implicated in *C9orf72* HRE in Chinese FTD patients (Table S1). Though *C9orf72* pathogenic expansion is quite rare in Chinese FTD patients, it is still necessary to screen *C9orf72* in Chinese FTD patients, especially those with FTD or ALS family history. Also, follow‐up efforts need to be strengthened when FTD patients carry common gene mutations of FTD and ALS. FTD patients with severe dementia might have difficulties in communicating with caregivers and are more likely to miss timely and proper treatment when attacked by ALS.

There are currently no FDA‐approved drugs for FTD, but several off‐label medications can be used to manage the symptoms of FTD. Selective serotonin uptake inhibitors and Trazodone proved to attenuate the neuropsychiatric symptoms.^16^ However, these atypical antipsychotics should be used slowly and cautiously in case of potential extrapyramidal and cognitive side effects. The pharmacologic treatment including acetylcholinesterase inhibitors and NMDA antagonists for Alzheimer’s disease are unlikely to benefit FTD patients. EGb 761^®^ has been recommended in multiple guidelines for the treatment of MCI and dementia, which could improve not only the cognitive performance but also the neuropsychiatric symptoms of the patients.^17^, ^18^ EGb 761^®^ might be a potential drug for early treatment of FTD, which needs further studies. Speech therapy may have some efficacy on language dysfunction. Moreover, antisense oligonucleotide target is considered a potential target for patients with *C9orf72* HRE.

Collectively, this is the first description of *C9orf72*‐related FTD patients manifesting both dementia and ALS‐like symptoms in mainland China, which broaden the genetic and clinical features of FTD.

## Conflict of interest

The authors declare that there is no conflict of interest.

## Consent to Participate

The patient provided written consent for participation.

## 1 Consent for Publication

The patient provided written consent for disclosure of medical information and images.

## Supporting information

Table S1
