# Supplementary material for: Identification of pathogenic C9orf72 hexanucleotide repeat expansion in a Chinese patient with frontotemporal dementia: A case report
Source: CNS Neurosci Ther. 2021 Mar 31;27(6):725–7. doi: 10.1111/cns.13639 (PMC8111495; doi:10.1111/cns.13639)
Supplement: Supplementary file 1 — Table S1 [file CNS-27-725-s001.docx]

**Supplementary Table 1. The frequency of *C9orf72* hexanucleotide repeat expansion in Chinese FTD patients.**

| **Study** | **Frequency** | **Familial cases/ sporadic cases** |
| --- | --- | --- |
| Che et al. (2017) | 0/82 (0%) | 0/82 |
| Tang et al. (2016) | 0/52 (0%) | 7/45 |
| Shi et al. (2016) | 0/38 (0%) | N/A |
| Lin et al. (2014) | 0/9 (0%) | N/A |
| Jiao et al. (2014) | 1/18 (5.6%) | 5/13 |
| This study | 1/37 (2.8%) | 5/32 |
| Total | 2/236 (0.8%) | N/A |

N/A: not available

**Reference**

1. Che XQ, Zhao QH, Huang Y, *et al.* Genetic Features of MAPT, GRN, C9orf72 and CHCHD10 Gene Mutations in Chinese Patients with Frontotemporal Dementia. Curr Alzheimer Res. 2017;14:1102-1108.

2. Tang M, Gu X, Wei J, *et al.* Analyses MAPT, GRN, and C9orf72 mutations in Chinese patients with frontotemporal dementia. Neurobiol Aging. 2016;46:235 e211-235.

3. Shi Z, Liu S, Xiang L, *et al.* Frontotemporal dementia-related gene mutations in clinical dementia patients from a Chinese population. J Hum Genet. 2016;61:1003-1008.

4. Lin CH, Chen TF, Chiu MJ, Lin HI, Wu RM. Lack of c9orf72 repeat expansion in taiwanese patients with mixed neurodegenerative disorders. Front Neurol. 2014;5:59.

5. Jiao B, Tang B, Liu X*, et al.* Identification of C9orf72 repeat expansions in patients with amyotrophic lateral sclerosis and frontotemporal dementia in mainland China. Neurobiol Aging. 2014;35:936 e919-922.
